# Ticks and tick-borne pathogens at the cutaneous interface: host defenses, tick countermeasures, and a suitable environment for pathogen establishment

**DOI:** 10.3389/fmicb.2013.00337

**Published:** 2013-11-19

**Authors:** Stephen Wikel

**Affiliations:** Department of Medical Sciences, Frank H. Netter MD School of Medicine, Quinnipiac UniversityHamden, CT, USA

**Keywords:** ticks, tick saliva, immune response, pathogen transmission, tick–host interface, immune modulation, skin

## Abstract

Ticks are unique among hematophagous arthropods by continuous attachment to host skin and blood feeding for days; complexity and diversity of biologically active molecules differentially expressed in saliva of tick species; their ability to modulate the host defenses of pain and itch, hemostasis, inflammation, innate and adaptive immunity, and wound healing; and, the diverse array of infectious agents they transmit. All of these interactions occur at the cutaneous interface in a complex sequence of carefully choreographed host defense responses and tick countermeasures resulting in an environment that facilitates successful blood feeding and establishment of tick-borne infectious agents within the host. Here, we examine diverse patterns of tick attachment to host skin, blood feeding mechanisms, salivary gland transcriptomes, bioactive molecules in tick saliva, timing of pathogen transmission, and host responses to tick bite. Ticks engage and modulate cutaneous and systemic immune defenses involving keratinocytes, natural killer cells, dendritic cells, T cell subpopulations (Th1, Th2, Th17, Treg), B cells, neutrophils, mast cells, basophils, endothelial cells, cytokines, chemokines, complement, and extracellular matrix. A framework is proposed that integrates tick induced changes of skin immune effectors with their ability to respond to tick-borne pathogens. Implications of these changes are addressed. What are the consequences of tick modulation of host cutaneous defenses? Does diversity of salivary gland transcriptomes determine differential modulation of host inflammation and immune defenses and therefore, in part, the clades of pathogens effectively transmitted by different tick species? Do ticks create an immunologically modified cutaneous environment that enhances specific pathogen establishment? Can tick saliva molecules be used to develop vaccines that block pathogen transmission?

## INTRODUCTION

Complexity of tick–host–pathogen interactions is reflected in over 900 tick species; broad range of vertebrate host species from which blood meals are successfully obtain, often by an individual tick species; tick transmission of the greatest variety of infectious agents of any blood feeding arthropod; increasing incidence and geographic occurrence of tick-borne diseases; and, long durations of host attachment, which require tick adaptations to cope with an array of host defenses (Jongejan and Uilenberg, 2004; Dennis and Piesman, 2005; Anderson and Magnarelli, 2008; Barker and Murrell, 2008; Brossard and Wikel, 2008; Randolph, 2010). Although all ticks are pool feeders, variations among species occur in depth of tick mouthpart penetration into host skin and production of attachment cement (Moorhouse, 1969). Those variations detected by histopathological studies of tick bite sites hinted at evolutionary variations in tick–host relationships, the vast scope of which is now becoming widely appreciated as a result of characterization of the salivary gland transcriptomes of many tick species (Ribeiro and Francischetti, 2003). During the past 40 years, tick–host–pathogen studies progressed remarkably from asking whether or not hosts developed adaptive immune responses to tick feeding to currently defining the molecular and cell biological nature of tick–host interactions (Willadsen, 1980; Wikel, 1982, 1996; Ribeiro, 1995; Ribeiro et al., 2006; Steen et al., 2006; Brossard and Wikel, 2008; Fontaine et al., 2011; Oliveira et al., 2011; Kazimirova and Stibraniova, 2013). Research activity continues to increase as indicated by a PubMed search of the term “tick salivary gland,” which revealed seven manuscripts published during 1973; 53 published during 2010; and, 31 published during the first 8 months of 2013.

## TICK MODULATION OF THE HOST ENVIRONMENT: SCOPE

Blood feeding evolved along multiple times along independent pathways among arthropods (Adams, 1999). Differences in duration of host interactions range from obtaining a blood meal within minutes in the case of mosquitoes to continuous attachment to the host for days in the case of ticks. Host defense mechanisms of the skin are a threat to successful blood feeding that must be counteracted. Arthropod saliva provides the answer to the challenges posed by redundant host defense mechanisms of the skin (Ribeiro and Francischetti, 2003; Francischetti et al., 2009). Tick saliva contains complex mixtures of molecules essential for obtaining a blood meal that inhibit hemostasis; block pain and itch responses that could alert an individual to the presence of an attaching or feeding tick; modulate angiogenesis and extracellular matrix remodeling related to wound healing; and, act as immunomodulators of innate and adaptive immunity (Wikel, 1996; Wikel and Bergman, 1997; Steen et al., 2006; Brossard and Wikel, 2008; Oliveira et al., 2011; Mans, 2011; Stibraniova et al., 2013). Tick saliva contains hundreds of different proteins that are differentially expressed during blood feeding (Ribeiro et al., 2006). Differences in salivary gland gene expression and saliva composition exist across and within genera while some gene families are conserved across both argasid and ixodid families of ticks (Alarcon-Chaidez et al., 2007; Mans et al., 2008; Francischetti et al., 2009; Kazimirova and Stibraniova, 2013). Likewise, non-protein molecules such as prostaglandin E2 and purine nucleoside adenosine are immunomodulatory molecules in tick saliva (Steen et al., 2006; Oliveira et al., 2011).

Variations in tick salivary gland transcriptomes and saliva composition represent adaptations to physiological differences among the broad range of vertebrate hosts and the continuous interplay between tick and host during blood feeding. Although host defenses may share common features across species, differences among host species likely result in evolutionary adaptations in saliva molecules for specific tick–host relationships. Likewise, variations during blood feeding in expression of gene family members would appear to be an effective strategy to reduce development of host immunity to saliva molecules important, or essential, for successful survival of the engorging tick. Depending upon tick mouthpart structure among ixodid species, saliva may be injected at the dermal–epidermal border or deep into the dermis (Moorhouse, 1969). Tick saliva is not simply injected into the skin in a hypodermic needle and syringe manner. Immunofluorescent microscopic examination of tick bite site skin biopsies revealed saliva antigens trapped in attachment cement; in all layers of the epidermis close to the site of tick attachment; and, at the dermal–epidermal border some distance from the mouthparts, suggesting the potential impact of saliva proteins over a wider area than might be anticipated (Allen et al., 1979). Complexity of events at the tick host interface is increased by the process in which injection of saliva occurs alternatingly with uptake of blood as well as of digested tissues at an increasing rate over the course of blood feeding.

Skin has long been recognized as a physical barrier providing protection from injury and infection. As a first line of defense, skin possesses an abundant population of cells and molecular mediators of innate and adaptive immunity, resulting in what is recognized as the skin immune system (Kupper and Fuhlbrigge, 2004; Nestle et al., 2009; Clark, 2010). Laceration of the skin immediately brings tick mouthparts into contact with keratinocytes, which are important immune sentinels possessing receptors of the innate immune response, anti-microbial peptides, and pro-inflammatory cytokines (Martinon et al., 2009; Nestle et al., 2009). The epidermis also contains important dendritic, antigen presenting, Langerhans cells (Merad et al., 2008), which take up tick saliva antigens and transport them to draining lymph nodes (Allen et al., 1979; Nithiuthai and Allen, 1985).

As mouthparts are thrust into the dermis, saliva comes into contact with nerve endings, blood and lymphatic vessels, fibroblasts, dendritic cells, macrophages, mast cells, natural killer (NK) cells, effector, and numerous long lived memory T lymphocytes, and soluble mediators such as complement, cytokines, chemokines, and lectins that contribute to local and systemic immune responses (Kupper and Fuhlbrigge, 2004; Nestle et al., 2009; Clark, 2010). Tick species developed multiple and diverse countermeasures to circumvent many aspects of each of these potential threats to increase their likelihood of successful host attachment and blood feeding. Tick-borne infectious agents, which themselves can modulate host immune defenses, are introduced into host skin where tick saliva reduced or modulated host defenses, resulting in an environment potentially more favorable for pathogen establishment and development (Wikel, 1999; Frischknecht, 2007).

Tick salivary gland gene expression profiles and saliva composition are topics of comprehensive reviews (Ribeiro and Francischetti, 2003; Steen et al., 2006; Anderson and Valenzuela, 2008; Brossard and Wikel, 2008; Mans et al., 2008; Francischetti et al., 2009; Fontaine et al., 2011; Mans, 2011; Oliveira et al., 2011; Kazimirova and Stibraniova, 2013; Stibraniova et al., 2013). A first line of defense is reducing the awareness of the attaching tick. Histamine and bradykinin are mediators of pain and itch responses induced as a result of tissue injury (Alexander, 1986; Schmelz, 2002, 2010). Saliva of selected tick species contain kininases (Ribeiro and Mather, 1998) and amine (histamine)-binding lipocalins (Paesen et al., 2000) capable reducing pain and itch responses, which could alert the host to the presence of an attaching and feeding tick.

Inhibiting the complex mechanisms of hemostasis is crucial to successful blood feeding and anti-hemostasis strategies evolved by ticks include saliva vasodilators, inhibitors of platelet aggregation, and molecules that delay or inhibit components of the coagulation cascade (Francischetti, 2010; Mans, 2011; Kazimirova and Stibraniova, 2013). Tick saliva derived vasodilators include non-protein prostaglandins (Ribeiro et al., 1992) and prostacyclin (Ribeiro et al., 1988). Tick saliva proteins contributing to vasodilation are histamine releasing factor (Dai et al., 2010) of *Ixodes scapularis* and a serine proteinase inhibitor (Chmelar et al., 2012) of *I. ricinus*. Tick saliva modulation of the coagulation cascade frequently focuses on factor Xa (Ibrahim et al., 2001; Narasimhan et al., 2002), including factor Xa activation of factor V (Schuijt et al., 2013); contact phase inhibition (Decrem et al., 2008); and, anti-thrombin activity (Koh and Kini, 2009). Tick saliva acts in multiple ways to inhibit platelets by hydrolyzing ADP; inhibiting collagen activation of platelet receptors and platelet aggregation; and, by its action on thrombin induced platelet aggregation (Francischetti, 2010). A diagrammatic representation of the potential interactions of host hemostasis and pain response pathways initiated by tick feeding is provided in Steen et al. (2006). A description of anti-hemostasis strategies across blood feeding arthropod families is provided with an excellent diagrammatic representation in the review by Fontaine et al. (2011).

Tick laceration of the skin, insertion of mouthparts, and creation of a “pool-like” feeding site in the dermis is an injury that would initiate the complex synchronized events of wound repair consisting of the immediate response, inflammation, proliferation, migration, contraction, and migration phases (Shaw and Martin, 2009). Wound healing is a challenge to a tick which might need to remain attached and obtain a blood meal for up to 2 weeks. Phases of wound healing are an excellent example of the interconnected nature of the defenses of hemostasis, inflammation, and immunity.

Hemostasis is an initial phase of wound healing, and as just described, it is extensively modulated by a variety of tick countermeasures. Inflammatory phase of wound healing involves influx and activation of neutrophils whose roles are to kill microbes and contribute to remodeling of extracellular matrix, formation of new blood vessels, and epithelia (Theilgaard-Monch et al., 2004). Ticks modulate neutrophil attraction by modifying chemokine and cytokine responses; reducing activation and down regulating reactive oxygen intermediates and nitric oxide; and, reducing surface integrin expression as well as endothelium expression of adhesion molecules (Brossard and Wikel, 2008; Kazimirova and Stibraniova, 2013). Wound healing is also modulated by tick saliva binding and inhibition of growth factors, reducing angiogenesis, impairing fibroblast migration, and by the presence of metalloproteases that remodel extracellular matrix (Fukumoto et al., 2006; Ribeiro et al., 2006; Kramer et al., 2008; Francischetti et al., 2009; Islam et al., 2009; Kazimirova and Stibraniova, 2013). Saliva binding and inhibition of transforming growth factor-beta (TGF-β), platelet-derived growth factor (PDGF), and fibroblast growth factor (FGF) reduces wound healing. These changes, when combined with reduction in angiogenesis, contributes to reduced likelihood of host rejection of the feeding tick.

Modulation of the inflammatory phase of wound healing by saliva molecules of multiple tick species has broader implications for impacting effector mechanisms of innate and adaptive immunity. Salivary gland extract of *Dermacentor andersoni* reduces endothelial cell expression of intracellular adhesion molecule-1 (ICAM-1) and a similar extract prepared from partially fed *I. scapularis* reduces vascular cell adhesion molecule-1 (VCAM-1) and P-selection (Maxwell et al., 2005). Although different adhesion molecules are targeted by different tick species, outcomes of potentially reducing leukocyte extravasation from the vascular compartment to the bite site and for endothelial cell antigen presentation are similar. Inflammatory influx is further impaired by *I. scapularis* saliva disintegrin metalloprotease-like saliva molecules, which down-regulate β2 integrin adhesion molecule expression on neutrophils (Guo et al., 2009). *I. scapularis* evolved saliva adaptations to suppress both leukocyte and endothelial cell molecules important in cell migration to sites of injury and infection.

Development of host innate and acquired immune responses to tick saliva components pose significant threats to an initial tick infestation occurring for several days and to subsequent infestations throughout the life of a host. Repeated infestation of guinea pigs and some cattle breeds with selected tick species induces immune mediated resistance that significantly reduces the number and blood meal volume of feeding ticks (Trager, 1939; Riek, 1962; Allen, 1973) while other tick–host associations do not result in acquired resistance to infestation (Brossard and Wikel, 2008).

Repeated exposure to tick bites induces responses that reduce pathogen transmission from tick to host. Rabbits infested with pathogen-free *D. andersoni* adults were significantly resistant to *Francisella tularensis* infection when subsequently infested with infected *D. andersoni* nymphs (Bell et al., 1979). Co-feeding transmission of Thogoto virus was reduced when infected ticks were fed on tick resistant guinea pigs (Jones and Nuttall, 1990). Repeated infestation with uninfected *I. scapularis* nymphs induced resistance to subsequent transmission of *Borrelia burgdorferi* by nymphs infesting guinea pigs expressing acquired resistant to tick feeding (Nazario et al., 1998) or mice not resistant to tick feeding (Wikel et al., 1997). A survey of human subjects enrolled in a 14-year study of Lyme disease risk in an endemic area revealed that individuals who experienced cutaneous hypersensitivity, itch, to tick bite were significantly less likely to develop *B. burgdorferi* infection (Burke et al., 2005). These studies suggest that tick feeding can induce host responses that diminish, modify, and/or neutralize the actions of saliva molecules, which may influence pathogen transmission. Potential contributing factors to this resistance are revealed by histological examination of *I. scapularis* nymph attachment sites on mice receiving a first infestation or a repeated infestation. Attachment site during an initial infestation contained very few inflammatory cells at the bite site, while similar sites on mice infested 1 month earlier and now receiving a second infestation, had a significant inflammatory infiltrate surrounding the tick mouthparts (Krause et al., 2009). Skin biopsies of *I. scapularis* attachment sites on tick infested people were histologically similar to those of infested mice (Krause et al., 2009).

Innate and adaptive immune responses are particularly significant threats to successful blood feeding to which different tick species developed broad and diverse arrays of immunomodulatory countermeasures (Gillespie et al., 2000; Valenzuela, 2004; Steen et al., 2006; Brossard and Wikel, 2008; Fontaine et al., 2011; Oliveira et al., 2011; Kazimirova and Stibraniova, 2013; Stibraniova et al., 2013). Diversity exists in regard to the capabilities of individual tick species to modulate specific elements of host immunity; however, collectively ticks evolutionarily developed efficient strategies to counteract essentially all of the major categories of immune defenses. Modulation of host immunity is especially essential for successful blood feeding, and as such provides an environment that facilitates pathogen transmission, establishment, and dissemination (Wikel, 1999; Brossard and Wikel, 2008; Nuttall and Labuda, 2008; Hovius, 2009).

Alternative, lectin and classical pathways of complement activation are primary lines of defense against infectious agents with the alternative and lectin pathways being integral to innate immune defenses (Ricklin et al., 2010). Complement deposition occurs at the dermal–epidermal junction of *D. andersoni* attachment sites on previously infested hosts (Allen et al., 1979). The alternative pathway is the most common target for tick modulation. Alternative pathway activation is an essential component for expression of host acquired resistance to *D. andersoni* infestation (Wikel, 1979). *I. scapularis* saliva inhibits C3b deposition and release of the anaphylatoxin C3a, which possesses both vasoactive and chemotactic activities (Ribeiro and Spielman, 1986; Ribeiro, 1987). Salivary gland extracts of *I. ricinus*, *I. hexagonus,* and *I. uriae* each inhibited alternative pathway activation in sera of natural host species (Lawrie et al., 1999). *I. scapularis* saliva dissociates factor Bb from C3b in the alternative pathway C3 convertase (Valenzuela et al., 2000; Tyson et al., 2007). *I. ricinus* saliva also inhibits alternative pathway activation (Daix et al., 2007) and binds factor P, whose function is to stabilize the alternative pathway C3 convertase (Couvreur et al., 2008). Blocking factor P binding allows complement regulatory proteins to displace C3b from factor Bb and cleave C3b, thus reducing alternative pathway activity. Salivary glands extract of the argasid tick, *Ornithodoros moubata*, inhibits C5a production from cleavage of C5 by both classical and alternative C5 convertases, inhibiting molecular assembly of the late steps of complement activation (Nunn et al., 2005). Targeted inhibition of the alternative pathway has significant implications for survival of tick-borne infectious agents deposited at the bite site.

Natural killer cells are innate immune response effectors capable of killing certain types of tumor cells and microbes; however, NK cells also function as regulatory cells in interactions with endothelial cells, dendritic cells, macrophages, and T lymphocytes (Vivier et al., 2008). Down-regulating the action of NK cells, while reducing an anti-microbial defense, would potentially reduce inflammation and impact the ability of the innate immune response to skew the subsequent adaptive immune response along a specific pathway. Salivary gland extracts prepared from *Dermacentor reticulatus*, *Amblyomma variegatum*, *Haemaphysalis inermis,* and *I. ricinus* each independently, moderately suppressed target cell killing (Kubes et al., 1994, 2002; Kopecky and Kuthejlova, 1998).

Neutrophils are the first wave of the innate immune response to injury and infection, and they occur in inflammatory infiltrates at tick attachment sites (Theis and Budwiser, 1974; Krause et al., 2009). As described for modulation of host wound healing, migration of neutrophils, and other leukocytes migration into tissues at tick attachment sites is modified by alteration of expression of adhesion molecule and their ligands on endothelial cells and leukocytes (Maxwell et al., 2005; Guo et al., 2009). Saliva of *I. scapularis* inhibits neutrophil phagocytosis and superoxide anion mediated killing (Ribeiro et al., 1990), and an *I. ricinus* salivary gland lipocalin reduces neutrophil activation and chemotaxis (Beaufays et al., 2008). Multiple tick species synthesize salivary gland inhibitors for the neutrophil chemoattractant interleukin-8 (IL-8, also known as CXCL8) and other chemokines (Hajnická et al., 2001; Vancová et al., 2010).

Through their role in orchestrating immune responses, skin dendritic cell subpopulations are a vital link between innate and adaptive immunity (Nestle et al., 2009), which makes them logical cell populations to be modulated by tick saliva. Epidermal Langerhans cells are dendritic cells (Merad et al., 2008) that trap tick saliva antigens and transport them to draining lymph nodes for presentation to T lymphocytes (Allen et al., 1979; Nithiuthai and Allen, 1985). Langerhans cells induce CD4^+^ lymphocyte differentiation to Th2 effector lymphocytes (Hunger et al., 2004). Many tick species preferentially induce host Th2 lymphocyte responses, which are thought to down regulate pro-inflammatory cytokines (Brossard and Wikel, 2008). Different immunomodulatory strategies are employed by different tick species. *Rhipicephalus sanguineus* saliva inhibits dendritic cell differentiation (Cavassani et al., 2005); reduces expression of chemokine receptor and migration in response to chemoattractants (Oliveira et al., 2008) and, increases production of interleukin-10 (Oliveira et al., 2010). *I. scapularis* saliva inhibits dendritic cell cytokine elaboration and expression of the co-stimulatory molecule CD40, resulting in reduced stimulation of CD4^+^ T lymphocytes (Sá-Nunes et al., 2007). *Rhipicephalus appendiculatus* saliva protein inhibits dendritic cell up-regulation of co-stimulatory molecule CD86 and maturation marker CD83; reduces secretion of the cytokines interferon-α (INF-α), interferon-γ (INF-γ), IL-1, IL-6, IL-12, and TNF-α; and, thus diminishes Th1 and Th17 polarizing ability of human monocyte derived dendritic cells (Preston et al., 2013).

Selected tick host relationships are characterized by basophil rich accumulations at tick attachment sites upon repeated infestation (Allen, 1973; Allen et al., 1977). Basophils have a non-redundant role in acquired resistance to *Haemophysalis longicornis* (Wada et al., 2010). Interestingly, basophils can act as antigen presenting cells, and they skew CD4^+^ T lymphocyte responses to a Th2 profile (Sokol and Medzhitov, 2010). Tick feeding preferentially induces Th2 polarization (Brossard and Wikel, 2008).

Cytokines are involved in nearly every aspect of immune regulation and effector function, and they represent a natural communication bridge between innate and adaptive immunity (Dinarello, 2007). Collectively, cytokines include interleukins, interferons, tumor necrosis factor, and chemokines. Many cytokines possess redundant and overlapping biological activities, including pro-inflammatory, anti-inflammatory, and immunoregulatory roles. In order to be successful blood feeding ectoparasites, diverse tick species developed mechanisms to modulate host interleukins, interferons, tumor necrosis factor, and chemokines; to suppress inflammatory responses; and, to deviate host T lymphocyte responses to profiles that are potentially less damaging to the feeding tick (Ramachandra and Wikel, 1992; Brossard and Wikel, 2008; Fontaine et al., 2011; Oliveira et al., 2011; Kazimirova and Stibraniova, 2013).

Tumor necrosis factor-α is a pro-inflammatory cytokine suppressed by salivary gland molecules of *D. andersoni* (Ramachandra and Wikel, 1992), *I. ricinus* (Pechová et al., 2004), *I. scapularis* (Hovius et al., 2008a), and *Hyalomma asiaticum asiaticum* (Wu et al., 2010). Simultaneous with suppression of TNF-α, *D. andersoni* salivary gland extract suppresses elaboration of the pro-inflammatory cytokines, IL-1 (Ramachandra and Wikel, 1992), IFN-γ (Prevot et al., 2009; Wu et al., 2010) accompanied by up-regulation of IL-10, which down-regulates Th1 cytokines, expression of MHC class II molecules, and macrophage co-stimulatory molecules (Pestka et al., 2004; Wu et al., 2010). Further tick modulation of cytokines occurs in the context of adaptive immune response modulation.

Chemokines are chemoattractant proteins with multiple, often redundant, roles in inflammatory and immune responses related to their elaboration and interactions with chemokine receptors that are differentially expressed on a variety of cell types (Sallusto and Baggiolini, 2008). Chemokine expression by endothelium and interaction with chemokine receptors on leukocytes modifies adhesion molecule interactions that facilitate extravasation of leukocytes across the vascular endothelium into tissues as well as activation at sites of injury and infection. Selective homing and localization of specific subpopulations of T lymphocytes to the skin are controlled by orchestrated, sequential, and integrated interactions of distinct sets of chemokines and adhesion molecules (Campbell et al., 1999; Sallusto and Baggiolini, 2008).

Tick modulation of chemokines, adhesion molecules, and pro-inflammatory cytokines constitutes an effective multi-faceted approach to down-regulation of host innate and adaptive immune responses that could negatively impact tick blood feeding success. Multiple chemokine inhibitory activities are associated with the saliva of numerous tick species (Brossard and Wikel, 2008; Kazimirova and Stibraniova, 2013). Salivary glands of *A. variegatum*, *R. appendiculatus*, and *R. sanguineus* contain inhibitors of the chemokine CCL3, which attracts neutrophils, monocytes, eosinophils, basophils, NK cells, and T lymphocytes (Vancová et al., 2007; Deruaz et al., 2008; Oliveira et al., 2008; Peterkova et al., 2008). Other chemokines blocked by tick salivary gland molecules include the neutrophil chemokine CXCL8 (IL-8); monocyte attractant CCL2; CCL4 attractant for monocytes and NK cells; CCL5 attractant for basophils, eosinophils, and T lymphocytes; CCL11 attractant for eosinophils; CCL18 attracting T lymphocytes; and, CXCL1 attracting neutrophils (Vancová et al., 2007; Deruaz et al., 2008; Oliveira et al., 2008; Peterkova et al., 2008). Tick anti-chemokine activities represent the evolution of saliva molecules that modulate multiple and redundant chemokine pathways.

Adaptive immune response modulation by ticks is achieved through the direct action of saliva molecules on B and T lymphocytes as well as through saliva induced changes on dendritic and other antigen presenting cells, and soluble mediators of the immune response (Ribeiro, 1995; Wikel, 1996; Gillespie et al., 2000; Steen et al., 2006; Brossard and Wikel, 2008; Oliveira et al., 2011; Fontaine et al., 2011; Kazimirova and Stibraniova, 2013; Stibraniova et al., 2013). Using a bioassay of cytokine function, T lymphocyte elaboration of the growth factor interleukin-2 (IL-2) was suppressed by *D. andersoni* salivary gland extract (Ramachandra and Wikel, 1992). An IL-2 binding protein was isolated from the salivary glands of *I. scapularis* (Gillespie et al., 2001). A commonly observed theme for numerous tick–host associations is infestation induced down regulation of the Th1 cytokines, IL-2 and IFN-γ, and up-regulation of the Th2 cytokine, IL-4, IL-5, IL-6, and IL-10 (Schoeler and Wikel, 2001; Brossard and Wikel, 2008; Kazimirova and Stibraniova, 2013). A novel 39.7 kDa sphingomyelinase-like protein cloned from an *I. scapularis* salivary gland cDNA library was the first tick protein shown to program host CD4^+^ T lymphocytes to express IL-4 (Alarcon-Chaidez et al., 2009).

Tick salivary gland molecules also inhibit T lymphocyte proliferation (Brossard and Wikel, 2008; Kazimirova and Stibraniova, 2013). A 36 kDa protein occurring in *D. andersoni* female and male saliva suppressed T lymphocyte proliferation (Bergman et al., 2000). A protein secreted by *I. ricinus* salivary glands suppresses T lymphocyte proliferation, induces a Th2 cytokine profile, and inhibits macrophage pro-inflammatory cytokines (Leboulle et al., 2002). The *I. scapularis* saliva protein, Salp15, inhibits IL-2 elaboration, CD4^+^ T lymphocyte proliferation, and T lymphocyte receptor intracellular signaling pathways (Juncadella and Anguita, 2009). Molecules similar to Salp15 likely occur widely in the genus *Ixodes* since homologues were found in the salivary glands of *I. ricinus* (Hovius et al., 2007), *I. pacificus* and *I. persulcatus* (Hojgaard et al., 2009). An *I. scapularis* saliva cystatin inhibited cytotoxic T lymphocyte proliferation *in vitro* and reduced an *in vivo* carrageenan induced inflammatory response (Kotsyfakis et al., 2006).

The Th17 lineage of CD4^+^ T lymphocytes are potent inducers of inflammatory responses (Korn et al., 2009). Transcriptional profiling of host *I. scapularis* attachment sites during initial and second infestations revealed an inhibition of Th17 immune responsiveness (Heinze et al., 2012). A gene encoding a salivary protein of *R. appendiculatus* was cloned, expressed, and the recombinant protein found to inhibit Th17 polarization (Preston et al., 2013). Concurrently with Th17 down-regulation, increased activity was observed for transcripts associated with regulatory T lymphocyte activity, IL-10, and suppressors of cytokine signaling molecules (Heinze et al., 2012).

## TICK MODULATION OF THE HOST ENVIRONMENT: IMPLICATIONS

The array of diverse strategies evolved by different tick species to modulate multiple, interconnected host defense pathways converge as a common outcome of providing an environment that favors tick feeding success and in turn provides an immunologically modified environment for establishment and dissemination of tick-borne infectious agents. Complexity of the co-evolution of ticks, tick-borne pathogens, and host defenses is just beginning to be dissected at the cell biological and molecular levels in combination with identification of tick saliva molecules responsible for modifying or driving many of these events. An amazing, orchestrated web of diverse interactions is being defined in this highly fertile research area.

Tick salivary gland molecules enhance transmission and establishment of Thogoto virus (Jones et al., 1989) and *Theileria parva* (Shaw et al., 1993) by *R. appendiculatus*; tick-borne encephalitis virus (Labuda et al., 1993) and *F. tularensis* (Krocova et al., 2003) by *I. ricinus*; vesicular stomatitis virus by *D. reticulatus* (Hajnická et al., 2000); and, *Borrelia burgdorfei* by *I. scapularis* (Zeidner et al., 2002) and by *I. ricinus* (Machackova et al., 2006; Horka et al., 2009). Influence of tick factors on the host response to *Borrelia* infection is demonstrated in development of a Th2 response component to *I. ricinus* transmission compared to a mixed Th1 and Th2 response following needle inoculation of 2 × 10^3^ spirochetes intradermally (Christe et al., 2000). *I. scapularis* infestation polarizes host cytokine responses to a Th2 profile and suppresses pro-inflammatory cytokines (Schoeler et al., 1999; Brossard and Wikel, 2008). Passively restoring pro-inflammatory (TNF-α) and Th1 (IL-2, IFN-γ) lymphocyte cytokines reduced by tick feeding, significantly enhances resistance to *I. scapularis* transmission of *B. burgdorferi* (Zeidner et al., 1996). Tick salivary gland molecules efficiently reduce inflammatory infiltrates at tick attachment sites by multiple mechanisms. Inoculation of *I. ricinus* saliva with *Borrelia garinii* spirochetes results in a reduced inflammatory response and number of cells accumulating in draining lymph nodes (Severinová, 2005; Kern et al., 2011). Suggestion of reduced cellular traffic from the bite site naturally leads to the impact of *I. ricinus* saliva molecules on dendritic cells and their reduced ability to optimally present *Borrelia* antigens (Slamova et al., 2011; Lieskovska and Kopecky, 2012). Immunoglobulin response to *B. burgdorferi* outer surface protein C (OspC) is reduced by a B lymphocyte inhibitory *I. ricinus* inhibitory protein (Hannier et al., 2003). Protection of *Borrelia* from antibody is also attributed to the action of *I scapularis* Salp15 and its *I. ricinus* homologue (Hovius et al., 2008b). Additional Salp proteins contribute to protection and transmission of *Borrelia* (Kazimirova and Stibraniova, 2013).

The relationship amongst tick, Lyme borreliosis spirochetes, and host complement is an intriguing example of co-evolutionary adaptations. *I. scapularis* and *I. ricinus* saliva inhibit alternative pathway C3 convertase activity by causing dissociation of C3b from Bb (Valenzuela et al., 2000; Daix et al., 2007; Tyson et al., 2007). Tick salivary lectin pathway inhibitor (TSPLI) present in saliva of *I. scapularis* reduces spirochete killing by inhibiting lectin pathway complement mediated activation on the surface of *B. burgdorferi* (Schuijt et al., 2011). In concert with tick modulation of host complement defenses, *B. burgdorferi* itself inhibits innate immune defenses, including complement activation (Singh and Girschick, 2004; Hovius, 2009). *Borrelia burgdorferi* binds complement regulator acquiring proteins that facilitate binding of factor H and factor H-like regulator molecules that inhibit C3b (Hellwage et al., 2001; Kraiczy et al., 2001; Stevenson et al., 2002). A recent review describes *B. burgdorferi* complement evasion strategies involving host regulatory proteins; spirochete production of membrane-bound mimics to prevent complement activation; and, use of tick proteins (de Taeye et al., 2013). The impact of tick modulation of the host environment is all the more striking since peak transmission of spirochetes doses not occur until approximately 48 h of blood feeding by *I. scapularis* nymphs (des Vignes et al., 2001). A different impact of tick modulation of the host environment is anticipated for tick-borne encephalitis virus, which is transmitted within minutes of the initiation of blood feeding (Alekseev and Chunikhin, 1990).

Murine bone marrow derived macrophages exposed to *I. scapularis* saliva during stimulation with *Anaplasma phagocytophilum* display reduced elaboration of IL-1β, TNF-α, IL-6, and IL-12p40 (Chen et al., 2012). *I. scapularis* saliva inhibits pro-inflammatory cytokines through both Toll-like and Nod-like signaling pathways. Likewise, TNF-α stimulated peripheral blood mononuclear cells were diminished in their ability to secret IL-8 when cultured in the presence of *I. scapularis* saliva (Chen et al., 2012). As an interesting example of tick and tick-borne pathogen co-adaptation, *A. phagocytophilum* infection induces up-regulation of α1, 3-fucosyltransferases in *I. scapularis* to increase tick cold hardiness (Neelakanta et al., 2010).

## CONCLUSION

Based upon findings reported in the literature to date, there exists an increasing body of data that links tick manipulation of host defenses with those strategies used and/or beneficial to the infectious agents transmitted to avoid host immunity. Tick manipulation of host defenses is one element in determining the clade of pathogens transmitted successfully by a specific tick species. Tick innate immune defenses are also critical factors in determining tick vector competence, and fortunately the availability of genome data and functional genomics tools are providing new insights into understanding these important components of tick vector biology (Hajdusek et al., 2013). A productive avenue of investigation will be blending studies that investigate the interrelationship among a tick transmitted infectious agent; tick innate immunity and barriers for pathogen development; tick modulation of host defenses; and, pathogen modulation of host defenses.

Development of tick vector blocking vaccines to prevent transmission of multiple different pathogens transmitted is a potentially feasible control strategy. However, achieving a vaccine that is highly effective for human use will likely require multiple antigens that target saliva molecules that act in an integrated manner to provide protection. The task of antigen identification, optimal formulation, and delivery will be complicated (Willadsen, 2008). Saliva molecules implicated in potentiating pathogen transmission are potential vaccine candidate immunogens (Ramamoorthi et al., 2005; Dai et al., 2010; Kotsyfakis et al., 2010; Schuijt et al., 2011).

Significant progress is being made in characterizing tick manipulations of host defense; the inter-relatedness of those modification to enhance tick feeding success; and, the implications of those changes for successful pathogen transmission and establishment in the host. A potentially highly productive approach to dissection of the complex interrelationships of tick, host, and tick-borne pathogen over the course of tick blood feeding would be to institute a systems biology approach to the characterization of gene expression in all three components of this dynamic and important relationship. The tools for molecular analysis and the computational power are at hand. In addition to gaining fundamental understanding of the relationship of tick, host and pathogen, novel control strategies for tick-borne diseases will almost certainly emerge.

## Conflict of Interest Statement

The author declares that the research was conducted in the absence of any commercial or financial relationships that could be construed as a potential conflict of interest.
